# Hospital costs of nosocomial multi-drug resistant *Pseudomonas aeruginosa* acquisition

**DOI:** 10.1186/1472-6963-12-122

**Published:** 2012-05-23

**Authors:** Eva Morales, Francesc Cots, Maria Sala, Mercè Comas, Francesc Belvis, Marta Riu, Margarita Salvadó, Santiago Grau, Juan P Horcajada, Maria Milagro Montero, Xavier Castells

**Affiliations:** 1 Health Services Evaluation and Clinical Epidemiology Department, Hospital del Mar-IMIM, Barcelona, Spain; 2 Preventive Medicine and Public Health Training Unit IMAS-UPF-ASPB, Barcelona, Spain; 3 CIBER de Epidemiología y Salud Pública (CIBERESP), Barcelona, Spain; 4 Ciencias de la Salut, Universitat Oberta de Catalunya, Barcelona, Spain; 5 Departament de Pediatria, Ginecología i Salut Pública, UAB, Barcelona, Spain; 6 Department of Microbiology, Laboratorio de Referencia de Catalunya, Barcelona, Spain; 7 Pharmacy Service, Hospital del Mar-IMAS, Barcelona, Spain; 8 Internal Medicine and Infectious Diseases Department, Hospital del Mar, Barcelona, Spain

**Keywords:** Antimicrobial resistance, Economic analysis, Hospital cost, Multi-drug resistance, Pseudomonas aeruginosa

## Abstract

**Background:**

We aimed to assess the hospital economic costs of nosocomial multi-drug resistant *Pseudomonas aeruginosa* acquisition.

**Methods:**

A retrospective study of all hospital admissions between January 1, 2005, and December 31, 2006 was carried out in a 420-bed, urban, tertiary-care teaching hospital in Barcelona (Spain). All patients with a first positive clinical culture for *P. aeruginosa* more than 48 h after admission were included. Patient and hospitalization characteristics were collected from hospital and microbiology laboratory computerized records. According to antibiotic susceptibility, isolates were classified as non-resistant, resistant and multi-drug resistant. Cost estimation was based on a full-costing cost accounting system and on the criteria of clinical Activity-Based Costing methods. Multivariate analyses were performed using generalized linear models of log-transformed costs.

**Results:**

Cost estimations were available for 402 nosocomial incident *P. aeruginosa* positive cultures. Their distribution by antibiotic susceptibility pattern was 37.1% non-resistant, 29.6% resistant and 33.3% multi-drug resistant. The total mean economic cost per admission of patients with multi-drug resistant *P. aeruginosa* strains was higher than that for non-resistant strains (15,265 vs. 4,933 Euros). In multivariate analysis, resistant and multi-drug resistant strains were independently predictive of an increased hospital total cost in compared with non-resistant strains (the incremental increase in total hospital cost was more than 1.37-fold and 1.77-fold that for non-resistant strains, respectively).

**Conclusions:**

*P. aeruginosa* multi-drug resistance independently predicted higher hospital costs with a more than 70% increase per admission compared with non-resistant strains. Prevention of the nosocomial emergence and spread of antimicrobial resistant microorganisms is essential to limit the strong economic impact.

## Background

Antibiotic resistance is a growing clinical problem and a major public health threat. Infections caused by antibiotic resistant microorganisms usually result in significantly higher morbidity, longer hospitalization, increased mortality rates, and excess health care costs compared with antibiotic-susceptible microorganisms [1,2]. Although much attention has focused on emerging antibiotic-resistant Gram-positive bacteria such as methicillin-resistant Staphylococcus aureus and vancomycin-resistant Enterococcus, resistance within Gram-negative bacilli continues to rise [3], occasionally creating situations in which few or no antibiotics that retain activity are available.

*Pseudomonas aeruginosa* is a Gram-negative bacterial pathogen that causes severe nosocomial infections [4]. This microorganism is a leading cause of nosocomial infections and is responsible for 10% of all hospital-acquired infections, ranking second among Gram-negative pathogens [5,6]. Multi-drug resistance to antipseudomonal antibiotics is a common and increasing problem in some hospitals [7,8]. Infections by multi-drug resistant *P. aeruginosa* (MDRPA) are associated with increased morbidity [9], mortality [7,10], and economic impact [11]. A number of studies have evaluated the impact of *P. aeruginosa* antibiotic resistance through assessment of in-hospital mortality rates and length of hospitalization [7-11]. However, fewer studies have quantitatively examined in detail the hospital economic impact of MDRPA infections and most have been limited to case series studies and outbreaks [11,12].

Not only morbidity and mortality, but also the economic cost may be a sensitive measure to quantify the burden of antimicrobial resistance [13]. In addition, estimating cost outcomes due to antimicrobial resistance may be useful to prompt hospitals and health care providers to introduce and support initiatives to prevent such infections, to influence health care providers to follow isolation guidelines, and to make appropriate use of antibiotics. Moreover, data can guide policy makers who take decisions on the funding of programs to track and prevent the spread of antimicrobial-resistant microorganisms.

The objective of this study was to assess the hospital economic costs of MDRPA acquisition in a tertiary hospital in Barcelona (Spain) during the period 2005–2006.

## Methods

### Study design and setting

A retrospective study was performed at Hospital del Mar, a 420-bed, urban, tertiary-care teaching hospital that covers an area of 300,000 inhabitants in the city of Barcelona, Catalonia (Spain). The study was carried out in compliance with the Helsinki Declaration. The study was approved by the ethics committee of IMIM-Hospital del Mar.

The study population consisted of all hospital admissions between January 1, 2005 and December 31, 2006 in which a nosocomial incident *P. aeruginosa* was isolated and identified. We used the microbiology laboratory records of the hospital to identify all inpatients with positive clinical cultures for *P. aeruginosa*. Clinical specimens were isolated and identified by the microbiology laboratory by means of routine techniques. Nosocomial incident *P. aeruginosa* acquisitions were defined as those in which the first *P. aeruginosa* positive culture for a particular patient occurred more than 48 h after patient admission during the study period. Overall, 410 incident nosocomial *P. aeruginosa* positive cultures were identified**.** The present report is based on 402 admissions with complete information on antibiotic susceptibility pattern and economical cost estimations (98% of the 410 positive cultures).

The susceptibility of isolates was determined by two methods: the MicroScan Walk away (Siemens Healthcare) (using NC36 and NC38 panels) or the Kirby Bauer method in Muller Hinton plates (Biomerieux Marcy l’etoile). Antibiotic susceptibility tests were performed using a standardized custom microtiter minimum inhibitory concentration (MIC). Testing procedures were validated by determining the MICs for reference strains. *P. aeruginosa* isolates were classified in three categories according to the antibiotic susceptibility pattern to all studied agents as: 1) non-resistant *P. aeruginosa* when the organism was susceptible to all the agents studied; 2) multi-drug resistant (MDRPA) for strains resistant to carbapenems, b-lactams, quinolones, tobramycin, and gentamicin and sensitive to colistin and amikacin [5,8,14]; and 3) resistant *P. aeruginosa* all the possible remainder combinations.

This information was linked to a hospital computerized patient records database. The following information was retrieved for each admission during the study period: patient age and sex, cause of hospitalization, medical treatments and comorbidities, previous hospitalization in the same hospital, previous intensive care unit (ICU) stay during the same hospital admission, exposure to invasive devices or procedures (mechanical ventilation, hemodialysis, bronchoscopy, digestive endoscopy and surgery); length of hospital stay (LOS) (including the LOS before detection defined as the period of time from admission to microorganism detection; and total LOS defined as the period of time from admission to the discharge or death of the patient), previous antibiotic therapy during the current hospitalization episode, and in-hospital mortality. Moreover, underlying illness severity was measured by the All Patient Refined Diagnosis Related Group Severity of Illness scale (version 20.0) [15].

### Costs estimation

The Municipal Institute of Health uses a hospital cost accounting system based on full-costing allocation that allows for assessing direct cost derived from clinical activity [16]. In the present study, cost estimation was based on a full-costing cost accounting system and on the criteria of clinical Activity-Based Costing (ABC) methods to obtain the highest sensitivity in the assessment of variability in clinical activity. Moreover, this system ensures that the hospital’s total costs are distributed among the patients. Allocation was based on directly assigning the cost of the following services to the patient: laboratory, pharmacy, radiology, nuclear medicine, pathology, and prosthetics [17,18]. The information systems contain exhaustive data on human resources and their activity: storage, admissions planning, ambulatory and emergency care, operating rooms, diagnostic and complementary tests, and inter-hospital consultations. This information creates and automatically updates the cost drivers for overheads [19,20].

The principal economic outcome assessed was total hospital cost of nosocomial MDRPA acquisition. To avoid the overestimation effect of costs due to extra LOS, all hospital costs were estimated once a positive culture was detected. This includes fixed cost, variable cost and pharmacy cost. Fixed cost derived from surgical procedures, hospitalization, and ICU stay, was distributed based on routine criteria: operation or intervention time, or the number of days of stay among the various hospitalization units. Variable cost included the costs derived from laboratory, radiology, pathology, prostheses, tests and pharmacy. In addition, we evaluated the effect of nosocomial MDRPA acquisition on pharmacy cost.

### Statistical analysis

Patient admission represented the unit of analysis. Univariate statistics were computed as customary. In contingency tables, Fisher's exact test for homogeneity or independence was applied to assess the relationship between two categorical variables; the Kruskal-Wallis and Mann–Whitney U-tests were used to compare continuous variables. The main effects of all predictors were independently explored in base models. Hospital costs were log transformed to achieve a normal distribution as far as possible. Multivariate analyses of hospital cost were performed using generalized linear models with the Gamma distribution and the log link function. The coefficients were converted to the measures of effect using an exponential transformation. Final multivariate models were adjusted for patient age and sex, comorbidities, average total cost per Diagnosis-Related-Groups (DRG) of patients with the lowest severity score, source of infection, stay (or not) in UCI after the infection and in-hospital mortality. The level of statistical significance was set at 0.05, and all tests were two tailed. Analyses were conducted using PASW Statistics 18 version 18.0.0 (IBM corp., 2009).

## Results

Overall, 402 incident *P. aeruginosa* positive cultures among hospitalized patients were identified during the study period. Their distribution by antibiotic susceptibility pattern was 149 (37.1%) non-resistant, 119 (29.6%) resistant, and 134 (33.3%) multi-drug resistant.

Table 1 shows the clinical characteristics of patients and the hospitalization process according to the antibiotic susceptibility pattern of *P. aeruginosa* isolates. The distribution of positive cultures differed between the sexes, with 67.2% and 74.6% of patients infected with resistant and multi-drug resistant PA, respectively, being male compared with 58.4% of patients with a non-resistant strain. The median age of patients was similar among all antibiotic susceptibility pattern groups. Patients with positive culture to resistant and multi-drug resistant PA strains showed a higher underlying illness severity score in comparison with those with non-resistant strains (41.2% and 45.0%, respectively, vs. 22.1%). Patients with resistant and multidrug-resistant PA strains were exposed in higher proportion to invasive procedures/devices, such as mechanical ventilation, hemodialysis, and bronchoscopy than patients with non-resistant strains. Resistant and multi-drug resistant PA strains were more prevalent among patients previously admitted to the ICU. The mean LOS was longer among patients with resistant (39.0 days) and multi-drug resistant *P. aeruginosa* (45.7 days) strains than among those with non-resistant strains (25.1 days). The use of previous anti-*P. aeruginosa* antibiotic therapy was more common among patients with resistant (45.4%) and multi-drug resistant *P. aeruginosa* (70.1%) isolates than in those with non-resistant isolates (13.4%). In-hospital mortality was significantly higher among patients with resistant and multi-drug resistant PA strains than in those with non-resistant strains (24.6% vs. 12.8%).

**Table 1:** **Principal characteristics for hospital admission according to the antibiotic susceptibility pattern of nosocomial*Pseudomonas aeruginosa*(PA) isolates. Barcelona (Spain), 2005–2006 (n = 402)**

	**Non-resistant PA**	**Resistant PA**	**Multi-drug resistant PA**	P^*^
	**N = 149 (37.1%)**	**N = 119 (29.6%)**	**N = 134 (33.3%) **	
**YEAR**				
2005	77 (51.7)	60 (50.4)	78 (58.2)	0.402
2006	72 (48.3)	59 (49.6)	56 (41.8)	
**PATIENT VARIABLES**				
**Male sex, n (%)**	87 (58.4)	80 (67.2)	100 (74.6)	0.015
**Age, years**				
Mean (sd)	68.6 (17.0)	67.4 (17.0)	67.4 (13.7)	0.348^**^
Median	71.0	73.0	70.5	
**Clinical conditions, n (%)**				
Chronic obstructive pulmonary disease	33 (22.1)	30 (25.2)	40 (29.9)	0.332
Solid neoplasia	41 (27.5)	25 (21.0)	27 (20.1)	0.289
Diabetes mellitus	26 (17.4)	28 (23.5)	23 (17.2)	0.364
Renal disease	15 (10.1)	5 (4.2)	19 (14.2)	0.021
Liver disease	10 (6.7)	10 (8.4)	17 (12.7)	0.216
HIV infection	6 (4.0)	4 (3.4)	4 (3.0)	0.942
Hematological neoplasia	5 (3.4)	4 (3.4)	4 (3.0)	1.000
Chemotherapy	1 (0.7)	1 (0.8)	3 (2.2)	0.531
**Source of infection**				
Respiratory	64 (42.9)	57 (47.9)	63 (47.0)	0.677
Skin & soft tissue	41 (27.5)	32 (26.9)	23 (17.2)	0.076
Genitourinary	26 (17.5)	15 (12.6)	27 (20.2)	0.275
Catheter	7 (4.7)	3 (2.5)	9 (6.7)	0.306
Endovascular	2 (1.3)	7 (5.9)	7 (5.2)	0.099
Abscess	3 (2.0)	5 (4.2)	1 (0.8)	0.184
Peritonitis	3 (2.0)	0 (0.0)	2 (1.5)	0.386
Digestive	2 (1.3)	0 (0.0)	1 (0.8)	0.779
Others	1 (0.7)	0 (0.0)	1 (0.8)	1.000
**Underlying illness severity score**				
1-3	112 (77.8)	62 (56.9)	68 (53.5)	<0.001
4	32 (22.1)	47 (41.2)	59 (45.0)	
**HOSPITALIZATION VARIABLES**				
**Previous hospitalization, n (%)**				
None	68 (45.6)	39 (32.8)	42 (31.3)	0.068
1	28 (18.8)	22 (18.5)	21 (15.7)	
2	20 (13.4)	20 (16.8)	20 (14.9)	
> = 3	33 (22.1)	38 (31.9)	51 (38.1)	
**Invasive devices or procedures, n (%)**				
Mechanical ventilation	9 (6.0)	29 (24.4)	31 (23.1)	<0.001
Hemodialisys	0 (0.0)	2 (1.7)	8 (6.0)	0.002
Bronchoscopy	13 (8.7)	12 (10.1)	23 (17.2)	0.080
Digestive endoscopy	12 (8.1)	6 (5.0)	12 (9.0)	0.479
Surgery	91 (61.1)	74 (62.2)	80 (59.7)	0.917
**Previous ICU stay (yes), n (%)**	18 (12.1)	40 (33.6)	34 (25.4)	<0.001
**LOS before detection, days**				
Mean (sd)	12.9 (10.0)	17.9 (15.5)	21.9 (14.9)	<0.001^**^
Median	10.0	14.0	18.0	
**Total LOS, days**				
Mean (sd)	25.1 (16.1)	39.0 (30.3)	45.7 (29.1)	<0.001^**^
Median	20.0	30.0	39.0	
**Previous antibiotic therapy, n (%)**				
None	69 (46.3)	25 (21.0)	15 (11.2)	<0.001
No anti-*Pseudomonas aeruginosa* drugs	60 (40.3)	40 (33.6)	25 (18.7)	
Anti-*Pseudomonas aeruginosa* drugs	20 (13.4)	54 (45.4)	94 (70.1)	
**In-hospital mortality, n (%)**	19 (12.8)	26 (21.8)	33 (24.6)	0.026

^*^Otherwise indicated p value derived form Fisher’s exact test. ^**^p value derived from Kruskal Wallis test.

HIV: Human Immunodeficiency Virus**;** ICU: intensive care unit; LOS: length of hospital stay.

The care of patients with antibiotic MDRPA positive cultures resulted in a significant higher median hospital economic cost than those with non-resistant strains (Table 2). The total mean economic cost per admission of patient with a MDRPA strain increased more than 3-fold in comparison with non-resistant strains (€15,265 vs. €4,933). This increased economic cost of resistant and multidrug-resistant *P. aeruginosa* strains was also observed in pharmacy, variable and fixed costs. The highest increased cost was found for pharmacy cost which was more than 6-fold higher in patients with MDRPA positive cultures in comparison with admissions with non-resistant strains (€2,781 vs. €473).

**Table 2:** **Economic costs (Euros) per hospital admission according to the antibiotic susceptibility pattern of nosocomial*Pseudomonas aeruginosa*(PA) isolates. Barcelona (Spain), 2005–2006 (n = 402)**

	**Non-resistant PA**	**Resistant PA**	**Multi-drug resistant PA**	P^*^
	**N = 149 (37.1%)**	**N = 119 (29.6%)**	**N = 134 (33.3%) **	
**Fixed cost**				
Mean (95% CI)	3,983 (3,168-4,797)	9,597 (7,184-12,009)	11,421 (8,858-13,983)	<0.001
Median	2,194 (1,673-2,629)	4,844 (3,809-6,860)	5,079 (3,922-7,024)	
**Variable cost**				
Mean (95% CI)	950 (701–1,200)	2,754 (1,504-4,005)	3,844 (2,742-4,946)	<0.001
Median	368 (250–523)	897 (670–1,502)	1,218 (812–1,867)	
**Pharmacy cost**				
Mean (95% CI)	473 (331–615)	1,964 (873–3,055)	2,781 (1,863-3,699)	<0.001
Median	135 (83–182)	594 (423–765)	725 (481–1,096)	
**Total cost**				
Mean (95% CI)	4,933 (3,911-5,956)	12,351 (8,858-15,845)	15,265 (11,834-18,696)	<0.001
Median	2,803 (2,026-3,431)	6,079 (4,829-8,049)	6,655 (4,771-10,200)	

^*^ p value derived from Kruskal Wallis test. CI: confidence interval.

On multivariate analysis, resistant and multi-drug resistant PA positive cultures were independently predictive of increased hospital total cost in comparison with non-resistant strains (an incremental increase in total hospital cost higher than 1.37-fold and 1.77-fold, respectively) (Table 3). In addition, other predictive factors independently contributed toward increased total hospital economic cost including ICU stay post infection and average cost per DRG (Table 3). After adjusting for potential confounders, the impact of MDRPA positive cultures was much higher on pharmacy cost (a 3.25-fold increase estimated with respect to non-resistant strains) than on total cost (Table 4).

**Table 3:** **Variables predicting total economic cost of nosocomial*Pseudomonas aeruginosa*isolates. Barcelona (Spain), 2005–2006, (N = 402)**

**Independent variable**	**Coefficient**	**(95% CI)**	***P***
**Antibiotic susceptibility pattern**			
Non-resistant	1.00		
Resistant	1.37	(1.08-1.72)	0.009
Multi-drug resistant	1.77	(1.41-2.22)	<0.001
**Sex, male**	1.12	(0.91-1.37)	0.277
**Age**	1.00	(0.98-1.00)	0.161
**Source of infection**			
Respiratory	0.91	(0.67-1.22)	0.538
Skin & soft tissue	0.85	(0.61-1.16)	0.311
Genitourinary	0.96	(0.67-1.36)	0.827
**Clinical conditions**			
Chronic obstructive pulmonary disease	0.79	(0.62-1.00)	0.045
Solid neoplasm	1.00	(0.79-1.24)	0.979
Diabetes mellitus	1.22	(0.96-1.55)	0.102
Renal disease	0.85	(0.61-1.16)	0.313
Liver disease	1.20	(0.87-1.65)	0.261
**UCI stay post infection**	3.46	(2.70-4.41)	<0.001
**Average total cost per DRG**	1.43	(1.28-1.60)	<0.001
**In-hospital mortality**	0.96	(0.75-1.21)	0.720

**Table 4:** **Influence of antibiotic susceptibility pattern of nosocomial*Pseudomonas aeruginosa*isolates on pharmacy cost. Barcelona (Spain) 2005–2006, (N = 402).**

	**Coefficient**	**(95% CI)**	***P***
Non-resistant	1.00		
Resistant	2.15	(1.54-2.99)	<0.001
Multi-drug resistant	3.25	(2.38-4.42)	<0.001

Adjusted for sex, age, average total cost per Diagnosis–related-groups, source of infection, clinical conditions, UCI stay post infection, and in-hospital mortality.

## Discussion

We assessed the hospital economic impact of nosocomial MDRPA acquisition in a tertiary hospital. Our comparison group was composed of patients with positive cultures admitted. Active surveillance of nosocomial infections was not implemented in our centre, thus most of the cases included in the present study corresponded with infected patients. Therefore, the present results should be interpreted as the impact of antibiotic resistance on hospital costs. The mean hospital total cost per admission was Є12,351 for patients who acquired resistant and €15,265 for MDRPA strains, which was substantially higher than the Є4,933 for those with non-resistant strains. On multivariate analysis, we found an independent contribution of *P. aeruginosa* multi-drug resistance acquisition to increased total cost per hospital admission, which was more than 1.7 fold higher in comparison with non-resistant microorganisms. Pharmacy cost showed the strongest increase, which was higher than 3-fold.

Antibiotic multi-resistant infections caused by Gram-negative bacilli are emerging in hospital settings and are becoming a major clinical and public health issue. Despite of the absence of an international consensus on the definition of multi-drug resistance in *P. aeruginosa* complicating comparison between studies [21], we found a higher prevalence of multi-drug resistance than previous reports from the European Antimicrobial Resistance Surveillance System (EARSS) Annual Report for 2006 http://www.rivm.nl/earss/result/Monitoring_reports/, 18% of *P. aeruginosa* isolates were found to be multidrug-resistant, i.e. resistant to three or more antibiotics from the EARSS protocol [5]. This change in trends strengthens the importance of assessing the impact of the increase in multi-drug resistance on hospital outcomes not only in terms of mortality or prolongation of hospital stay, but also on economic costs.

Globally, it is estimated that infections due to antimicrobial-resistant organisms have higher costs ranging between $6,000 and $30,000 than do infections due to antimicrobial susceptible organisms [13]. In our study, the mean cost per hospital admission with a positive culture of MDRPA was €15,265, which was substantially higher than that of hospital admissions infected with non-resistant strains (mean of €4,933). Previous studies have estimated the hospital cost increase due to MDRPA infections. In an ICU outbreak, Bou et al. [12] reported that the emergence of antibiotic multi-resistance in *P. aeruginosa* infections was significantly associated with a high median economic cost (€72,356 vs €17,090; P < 0.004). Lautenbach et al. [22] reported that patients infected with imipenem-resistant *Pseudomonas aeruginosa* had greater hospital costs (US$81,330 vs. US$48,381; P < 0.001). In a matched cohort study Carmeli et al. [9] estimated that emergence of resistance to *P. aeruginosa* increased the total hospital charges by $7,340. In a case-series study, Harris et al. [11] reported that the mean cost of the admission during which the first multi-resistant isolate of *P. aeruginosa* was cultured was of $54,081, compared with a mean cost of $22,116 for patients infected with susceptible strains. Most of these studies were limited to case series and outbreaks among patients in ICU, which may partly explain the differences in cost estimations in comparison with current estimations. Also the inclusion in our study of both infections and colonizations could result in underestimated costs. In addition, diverse cost estimation methodology could influence differences in cost estimations when comparing studies.

When analyzing different types of hospital cost, we found a remarkable increment in hospital cost due to MDRPA strains in pharmacy cost. Although not exclusively, pharmacy excess costs associated with multi-drug resistant microorganisms may partly be due to delayed appropriate antibiotic therapy and the need to use more expensive antibiotics, which implies a substantial increase in drug therapy cost. In our experience, colistin (polimixin-E) is the first choice antimicrobial for MDRPA infections [14]. Comparatively with other antipseudomonals colistin is as expensive as other broad- spectrum anti-pseudomonas antibiotics such as carbapenems or the pip/tazo combination. Importantly, these excess costs are potentially avoidable.

Notable among the strengths of the study is that information was available from almost all *P. aeruginosa* acquisitions detected among hospital admissions during the study period. This study has some limitations. To estimate the effect of antibiotic resistance on the economic cost of nosocomial acquisitions, patients with antibiotic resistant microorganisms were compared with those without resistant organisms for hospital cost outcomes. The problem with this simple comparison is that sicker patients with longer length of hospital stay, greater severity, and comorbidities are more likely to acquire resistant organisms, leading to substantial confounding bias. However, we addressed this problem by detailed adjustment to control as much as possible the factors other than antibiotic resistance that contributed to adverse outcomes, such as severity, and UCI stay post infection. This enabled us to isolate the independent effect of *P. aeruginosa* acquisition on hospital costs. Another relevant limitation in estimating hospital cost in relation to infection acquisition involves time and length bias [23]. However, no statistical solution exists for models involving a quantitative dependent variable such as cost, which cannot be directly related to time. Thus, to address this methodological issue we used costs after infection as dependent variable, which can reduce cost overestimation due to time and length bias. On the other hand, using generalized linear models (GLM) to estimate hospital costs could result in biased and over-estimated costs. However, a recent study comparing different analytic approaches did not demonstrate that multistate methods are better than GLM or propensity score adjustments [24]; and the most important issue in calculating the cost or the economic impact relies on using the real cost for each day of stay and for each patient and on the quality of the data [24]. Moreover, our cost estimates most closely represent the direct hospital perspective, which provides a partial view of the health care impact of antibiotic resistance. Thus, we believe that the hospital perspective is to some extend also underestimated because we did not account for extra costs to the institution at large attending patients with MDRPA infections, such as surveillance cultures, isolation supplies, and loss of beds due to the need to isolate a patient. And in addition, the effects extending beyond hospitalization were underestimated by this study. Finally, data from one centre and one particular health care system could limit broader applicability of the present results.

## Conclusions

In conclusion, MDRPA acquisition is becoming a serious issue in hospital settings and is independently associated with a substantial increase in medical costs. Optimal use of existing antimicrobial agents through education of health professionals, antibiotic policies (including antibiotic stewardship), implementation of infection control measures (e.g., hand hygiene, environmental cleaning, active screening cultures and isolation), and reducing the length of patient stay should be the strategies aimed at preventing the emergence and spread of antibiotic resistance in order to limit their adverse consequences, including their economic cost.

## Abbreviations

CI, Confidence interval; DRG, Diagnosis-Related-Groups; HIV, Human Immunodeficiency Virus; ICU, intensive care unit; LOS, length of hospital stay; MDRPA, multi-drug resistant P. aeruginosa; PA, Pseudomona aeruginosa.

## Competing interests

The authors declare that they have no competing interests.

## Authors’ contributions

EM carried out the statistical analyses and drafted the manuscript. MC and FB contributed statistical advice for analyses. MS, SG were involved in data collection and laboratory analysis. FC, MS, MR and XC participated in the design and coordination of the study and helped to draft the manuscript. JPH and MMM helped to draft the manuscript. All authors read and approved the final manuscript.

## Pre-publication history

The pre-publication history for this paper can be accessed here:

http://www.biomedcentral.com/1472-6963/12/122/prepub
